# Identifying environmental variables explaining genotype-by-environment interaction for body weight of rainbow trout (*Onchorynchus mykiss*): reaction norm and factor analytic models

**DOI:** 10.1186/1297-9686-46-16

**Published:** 2014-02-26

**Authors:** Panya Sae-Lim, Hans Komen, Antti Kause, Han A Mulder

**Affiliations:** 1 Animal Breeding and Genomics Centre, Wageningen University, P.O. Box 338, 6700, AH, Wageningen, The Netherlands; 2 MTT Agrifood Research Finland, Biotechnology and Food Research, Biometrical Genetics, FI-31600 Jokioinen, Finland; 3 Current address: Nofima, Osloveien 1, P.O. Box 210, NO-1431 Ås, Norway

## Abstract

**Background:**

Identifying the relevant environmental variables that cause GxE interaction is often difficult when they cannot be experimentally manipulated. Two statistical approaches can be applied to address this question. When data on candidate environmental variables are available, GxE interaction can be quantified as a function of specific environmental variables using a reaction norm model. Alternatively, a factor analytic model can be used to identify the latent common factor that explains GxE interaction. This factor can be correlated with known environmental variables to identify those that are relevant. Previously, we reported a significant GxE interaction for body weight at harvest in rainbow trout reared on three continents. Here we explore their possible causes.

**Methods:**

Reaction norm and factor analytic models were used to identify which environmental variables (age at harvest, water temperature, oxygen, and photoperiod) may have caused the observed GxE interaction. Data on body weight at harvest was recorded on 8976 offspring reared in various locations: (1) a breeding environment in the USA (nucleus), (2) a recirculating aquaculture system in the Freshwater Institute in West Virginia, USA, (3) a high-altitude farm in Peru, and (4) a low-water temperature farm in Germany. Akaike and Bayesian information criteria were used to compare models.

**Results:**

The combination of days to harvest multiplied with daily temperature (Day*Degree) and photoperiod were identified by the reaction norm model as the environmental variables responsible for the GxE interaction. The latent common factor that was identified by the factor analytic model showed the highest correlation with Day*Degree. Day*Degree and photoperiod were the environmental variables that differed most between Peru and other environments. Akaike and Bayesian information criteria indicated that the factor analytical model was more parsimonious than the reaction norm model.

**Conclusions:**

Day*Degree and photoperiod were identified as environmental variables responsible for the strong GxE interaction for body weight at harvest in rainbow trout across four environments. Both the reaction norm and the factor analytic models can help identify the environmental variables responsible for GxE interaction. A factor analytic model is preferred over a reaction norm model when limited information on differences in environmental variables between farms is available.

## Background

Body weight at harvest is an economically important trait in rainbow trout (*Onchorynchus mykiss*) and other farmed fish species. Rainbow trout can be produced in a wide range of farming environments. When genotype-by-environment interaction (GxE) is present and when selection is practiced only in a breeding environment, lower-than-expected genetic gains can be obtained in other production environments. Optimization of a breeding program to account for GxE interaction can increase genetic gain across environments [1-3]. Optimization may be expensive, for instance when environment-specific breeding programs need to be established. Alternatively, if environmental variables (EV) are changed so that they are similar across production environments, GxE interaction may decrease. This requires that the EV that cause GxE interaction are identified, which can be done by using a reaction norm model to quantify GxE interaction as the function of specific EV [4-6]. Alternatively, in a two-step factor analysis, a latent common factor responsible for GxE interaction is first identified and, subsequently, correlations between the common factor and EV are calculated to identify the significant EV [7]. In this study, our aim was to identify the EV that cause a strong GxE interaction for body weight at harvest in rainbow trout using a reaction norm model and a factor analytic model.

## Methods

### Data

The data used in this study were from a GxE experiment conducted in four different environments on three continents (North America, South America, and Europe as described by Sae-Lim et al. [8]). In August 2009, 100 full-sib families were produced from 58 sires and 100 dams (1 to 1.7 mating ratio) at the Troutlodge breeding company in Washington State (nucleus: NUC). Procedures for the ethical treatment of animals at Troutlodge, Inc. followed the US and/or State guidelines for animal care and use including those outlined by “Guidelines for Use of Fishes in Field Research” established by the American Fisheries Society (AFS), the American Society of Ichthyologists and Herpetologists (ASIH), and the American Institute of Fisheries Research Biologists (AIFRB). The same standard was applied for all animals in the study. Fertilization took place during a period of four weeks. Different water temperatures were used to synchronize embryonic development and hatching. At least 25 eyed eggs per family were shipped to each of the following three locations: (1) the re-circulating aquaculture system at the Freshwater Institute, Virginia, USA (FI); (2) a high altitude farm with low oxygen dissolved in water (Titicaca Lake) in Peru (PE); and (3) a low water temperature farm in Germany (GE). A random sample of 25 eyed eggs per family was maintained at NUC as a control. All fish were measured for body weight at harvest (BWH, in grams), in June 2010 (NUC), in July 2010 (FI), in August 2010 (PE), and in December 2010 (GE) (Table 1).

**Table 1:** Means and standard deviations (SD) for body weight at harvest (BWH) in four environments and means of environmental variables during the rearing period

**Environment**	**N**	**BWH (g)**	**SD (g)**	**Age (day)**	**Temp (°C)**	Day*Degree (day*°C)	**Oxygen (mg/L)**	**Photoperiod (min)**
NUC	2367	546.7	94.7	287.5	13.8	3940	7.3	223.1
FI	1893	395.2	75.8	294.0	12.5	3686	10.5	163.3
PE	2897	524.1	105.0	357.0	13.4	4805	6.6	−53.1
GE	1819	376.4	81.7	444.0	9.9	4439	12.0	292.9

N = number of observations; NUC = breeding environment; FI = Freshwater Institute; PE = Peru; GE = Germany; units are indicated between brackets.

### Pedigree reconstruction

The fish were tagged using passive integrated transponders (PIT tag; Allflex USA, Inc. for NUC, FI and PE, and DORSET Identification b.v., the Netherlands, for GE) and the PIT tag was scanned (scanner SF2001ISO: Destron Fearing, USA for NUC, FI and PE, and GR250: DORSET Identification b.v., the Netherlands, for GE) at an average size of 26.3 to 33.2 g (five to seven months of age). Before tagging, fish were anesthetized with MS222 (150 mg/L) in the NUC, FI, and PE farms and with clove oil (10 mg/L) in the GE farm. Fin clips were collected from all 158 parents and from the fish at tagging from FI, PE and GE for DNA extraction. In the NUC farm, fin clips were not collected, because fish were kept in separate full-sib family tanks until tagging, allowing the pedigree to be recorded.

DNA was isolated from fin clips to reconstruct pedigrees. Genotyping was done at three laboratories: National Center for Cool and Cold Water Aquaculture, USDA; Troutlodge, Inc.; and Animal Breeding and Genomics Centre, Wageningen University. The protocols for DNA isolation and genotyping were synchronized across the three laboratories. DNA isolation was done using the Nucleospin® 96 Tissue Core Kit. Multiplex PCR amplification was as described in [9]. Nine microsatellite markers were used for PCR: OMM1008, OMM1051, OMM1088, OMM1097 [10], OMM5007, OMM5047 [11], OMM5233, OMM5177 [12], and OMM1325 [13]. Multiplex PCR amplifications, i.e. quadroplex and pentaplex, were as follows [9]: an initial 5 min denaturation at 95°C, followed by 35 cycles of 30 s denaturation at 95°C, 45 s annealing at 55°C, and 90 s extension at 72°C, and a final 10 min extension step at 72°C. Fragment analysis of the PCR products was done by setting the fragment sizes to Genescan LIZ 500 size standard (Applied Biosystem). Output data were analysed using Genemapper software version 4 (Applied Biosystem) [14].

Parental allocation was performed using PAPA software [15] based on the known mating data to increase the accuracy of parental assignments [8]. In total, 2142 out of 2243 fish sampled in FI, 3106 out of 3236 fish sampled in PE, and 2104 out of 2235 fish sampled in GE were successfully allocated to the 100 full-sib families. The 362 fish that were not successfully allocated to a family were removed from the dataset. In total, six generations of pedigree information, one from the DNA reconstructed pedigree and five from the previous generations of pedigree information, were used in the genetic analyses.

### Environmental variables

Summary statistics of the EV are in Table 1.

#### Temperature

The average water temperature (°C) was measured in the tank (NUC, FI), raceway (GE) or lake (PE) during the rearing period of the experiment. In NUC, the average ambient temperature was between 13 and 14°C throughout the growing season. In FI, PE, and GE, the water temperature followed the natural (daily and seasonal) fluctuations. Water temperature was recorded every 15 min using a data logging Transmitter SC100 (Hach Lange, Germany) in NUC and GE. In FI, temperature was measured once a day using either a Hach HQ40d hand held meter or a SC100 Universal Controller (Hach Company, Loveland, CO). In PE, temperatures were measured with a standard mercury thermometer in the Titicaca Lake once a day for only a short period (September 3 to 16, 2010). However, water temperature of the Titicaca Lake does not fluctuate much throughout the year and varies between 12 and 14°C.

#### Age

Average age at harvest (in days) corresponded to the period between hatching and day of harvest. Differences in age at harvest were caused by differences in preferred market sizes across environments. In NUC, harvest was done twice (at 2 week intervals).

#### Day*Degree

In salmonids, the growth rate depends on the water temperature. The product of days to harvest and daily temperature is therefore commonly used in salmonid farming to compare days to harvest across temperature regimes. Day*Degree was calculated as: average water temperature during the growing period multiplied by average age at harvest.

#### Oxygen

The amount of oxygen dissolved in the water during the rearing period, recorded in mg/L or ppm, was calculated based on the average of daily measurements. In NUC, oxygen was measured daily in the morning (between 7:30 and 9:00 am) using a YSI model 550 (YSI, Yellow Springs, OH) at the inlet and outlet of the rearing tanks. In FI, oxygen was measured at a single position in the circular tanks once a day between 8:00 and 9:30 am, using a Hach HQ40d with a Hach LDO probe attachment, or a SC100 Universal Controller (Hach Company, Loveland, CO). In PE, dissolved oxygen was measured in the net pens of Titicaca Lake in the morning (between 9:00 and 10:00 am) for a short period of time (same as temperature), using the Hach dissolved oxygen test kit (Hach Company, Loveland, CO). In GE, dissolved oxygen level was controlled to be above 10 mg/L. When the dissolved oxygen decreased, supplementary oxygen was automatically released until the dissolved oxygen was above 10 mg/L. Dissolved oxygen was measured every 15 min using a data logging Transmitter SC100 (Hach Lange, Germany).

#### Photoperiod

**Figure 1:**
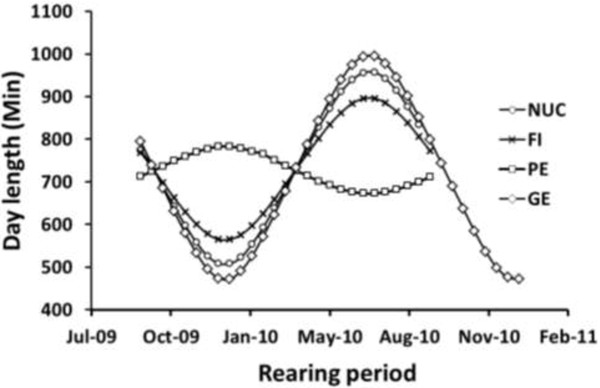
**Day length profiles in four experimental environments.** The x-axis represents the rearing period in two-month intervals (month-year); each observation represents the average day length during a two-week interval; the rearing period differed across environments: NUC = breeding environment, FI = Freshwater Institute, PE = Peru and GE = Germany.

Since the experiment was conducted across continents, changes in day length differed. “Photoperiod” was defined as the difference between the maximum day length observed during the rearing period and average day length during the rearing period. This measurement reflects the amplitude of day length, which provides more information than average day length. The locations that were used to calculate photoperiod were: Seattle in Washington State (NUC), Martinsburg in West Virginia (FI), Juliaca in Peru (PE), and Leipzig Schkeuditz in Germany (GE). Data on times of sunrise and sunset each week in 2009 and 2010 were obtained from http://www.wunderground.com/history/. Average day length was calculated as the difference between sunrise and sunset in minutes, for each day of the week in the rearing period (Figure 1). To account for differences between northern and southern hemispheres (NUC, FI and GE versus PE), we used negative and positive signs to indicate the directions of change in the photoperiod.

### Statistical analysis

In a previous study, we reported a significant GxE interaction for body weight at harvest in rainbow trout that were reared on three different continents [8]. Genetic correlations (0.19 to 0.48) were estimated using a multivariate model (multi-trait multi-environment) with correction for selection bias due to selective mortality. The same data were used to identify the EV that contributed to the GxE interaction in this study. ASReml was used for all models in this study.

#### Multivariate model

In this study, we compared reaction norm and factor analytic models with the multivariate model without selection bias correction. The multivariate model without the selection bias correction was:

$$Y_{\mathrm{hij}}=\mu +\beta_{h}\mathrm{AG}E_{h}+\mathrm{FER}T_{\mathrm{hi}}+a_{\mathrm{hj}}+e_{\mathrm{hij}},$$

where *Y*_*hij*_ is the observation (body weight at harvest) of the *j*^th^ individual in a given environment (*h* =1: NUC, 2: FI, 3: PE, and 4: GE), *μ* is the overall mean. *β*_*h*_ is the coefficient of linear fixed regression on age at harvest (*AGE*_*h*_) within the *h*^th^ environment. The (*AGE*_*h*_) was included in the model to correct for different measurement dates, and corrected for the length of the rearing period from hatching to the day of trait measurement. The orthogonal polynomial of (*AGE*_*h*_) was tested for significance up to the third order but the quadratic and the cubic orders were not significant based on a Wald test. *FERT*_*hi*_ is the effect of the *i*^th^ fertilization period within the *h*^th^ environment due to different groups of available fertile dams. *a*_*hj*_ is the random additive genetic effect, *a* ∼ MVN[**0**, **A** ⊗ **G**], of the *j*^th^ animal, where MVN is the multivariate normal distribution, **A** is the additive genetic relationship matrix among individuals and **G** is the additive genetic (co)variance matrix among body weight in the different environments. Residual covariances of the same trait measured in different environments were set to zero, because animals were measured in only one environment:

$$\mathrm{VAR}\;\left(e\right)=\left[\begin{array}{cccc} \sigma_{e_{1}}^{2} & 0 & 0 & 0\\ 0 & \sigma_{e_{2}}^{2} & 0 & 0\\ 0 & 0 & \sigma_{e_{3}}^{2} & 0\\ 0 & 0 & 0 & \sigma_{e_{4}}^{2} \end{array}\right],$$

where $\sigma_{e_{h}}^{2}$ is residual variance of body weight in different *h* environments.

#### Reaction norm model

The EV causing GxE interaction can be identified by fitting each EV in a reaction norm model. Random regression was used to estimate (co)variance components. The random animal effect was modelled as a function of the EV. The random regression model was:

$$Y_{\mathrm{hij}}=\mu +\eta_{h}+\beta_{h}\mathrm{AG}E_{h}+\mathrm{FER}T_{\mathrm{hi}}+\sum_{k=0}^{m}\alpha_{\mathrm{kj}}P_{\mathrm{kh}}+e_{\mathrm{hij},}$$

where *η*_*h*_ is fixed environmental effect (*h* =1: NUC, 2: FI, 3: PE, and 4: GE), accounting for different levels of environment and *α*_*kj*_ is random regression coefficient *k* for animal *j* for the orthogonal polynomial *P*_*kh*_ for an EV in environment *h,* with *m* maximum order of the polynomial. The matrix of random regression coefficients was assumed to be distributed multivariate normal: $\left[\begin{array}{c} \alpha_{0}\\ \vdots \\ \alpha_{m} \end{array}\right]∼\mathrm{MVN}\;\left[0,A⊗G_{\mathrm{RN}}\right]$, where MVN is the multivariate normal distribution, **A** is the additive genetic relationship matrix, and **G**_**RN**_ is the n*n genetic (co)variance matrix for parameters of the reaction norm model. The *n* is the highest order of polynomial (*m*) + 1. Residual effects *e*_*hij*_ for animal *j* in environment *h* were assumed distributed

$e∼N\left(0,\left[\begin{array}{cccc} I\sigma_{e_{1}}^{2} & 0 & 0 & 0\\ 0 & I\sigma_{e_{2}}^{2} & 0 & 0\\ 0 & 0 & I\sigma_{e_{3}}^{2} & 0\\ 0 & 0 & 0 & I\sigma_{e_{4}}^{2} \end{array}\right]\right)$, where **I** is the identity matrix. A third order polynomial for the random reaction norm model results in a 4×4 **G** matrix, which is the same dimension as the original multivariate model with four environments. When each environment has just one value for the environmental variable, the multivariate model and the reaction norm model yield identical genetic correlations [16]. Therefore, even meaningless EV would give the same results as the multivariate model. Thus, to identify EV responsible for the GxE interaction, we decided to use a first order polynomial (*m* = 1), because it is the simplest and has the largest difference in number of estimated parameters. The additive genetic variance ${\hat{V}}_{A}$ of BWH for each level of an EV was calculated as ϕ^'G^RNϕ^**,** where $\hat{\phi }$ is a n*1 vector of polynomial coefficients for each level of the EV and ϕ^' is the transposed vector of $\hat{\phi }$. The covariance (COV) between BWH at levels *i* and *j* of an EV was calculated as $\hat{\phi }{'}_{i}{\hat{G}}_{\mathrm{RN}}{\hat{\phi }}_{j},i\neq j$. The genetic correlation (*r*_g_) between BWH at levels *i* and *j* of an EV was calculated as $\frac{\mathrm{COV}\left(A_{EV_{i}},A_{EV_{j}}\right)}{\sqrt{{\hat{V}}_{A,EV_{i}}\times {\hat{V}}_{A,EV_{j}}}}$. Standard errors of estimates of genetic correlations were approximated with ASReml [17]. The sire BLUP-estimated breeding value (EBV) for each level of EV was calculated as $\hat{H}\hat{\phi }$, where $\hat{H}$ is a 1*n vector of sire BLUP-EBV for BWH*.* The sire BLUP-EBV of eight randomly selected sires were plotted against photoperiod as an example to show the degree of heterogeneity of additive genetic variance and re-ranking of sires, depending on change across levels of photoperiod.

#### Factor-analytic model

The factor analytic (FA) model identifies latent common factors that explain the variation in the data and can be used to estimate GxE interaction [18]. The FA animal mixed model was:

$$\begin{array}{l} Y_{\mathrm{hij}}=\mu +\eta_{h}+\beta_{h}\mathrm{AG}E_{h}+\mathrm{FER}T_{\mathrm{hi}}+A_{C_{j}}+A_{S_{\mathrm{hj}}}\\ \;\;+e_{\mathrm{hij}}, \end{array}$$

where $A_{C_{j}}$ is the random genetic effect of the latent common factor across environments for animal *j* and $A_{S_{\mathrm{hj}}}$ is the random genetic effect specific to environment *h* for animal *j*. The five genetic effects (one across environments and four specific to each environment) were assumed distributed multivariate normal: $\left(A_{c},A_{s_{1}},A_{s_{2}},A_{s_{3}},A_{s_{4}}\right)∼\mathrm{MNV}\left[0,A⊗G_{\mathrm{FA}}\right]$, where **A** is the additive genetic relationship matrix and **G**_**FA**_ is the genetic (co)variance matrix for common and specific animal effects. The common animal effect can be interpreted as the breeding value for the latent common factor whereas the specific animal effects are the environment-specific remnant breeding value unexplained by the common factor. Therefore, each animal has five breeding values. The genetic (co)variance matrix **G**_**FA**_ = **ΓΓ** ' + **Ψ**, where **Γ** is the matrix of factor loadings (coefficient vector of the latent common factor) and **Ψ** is the diagonal matrix of specific variances (*ψ*_*h*_) for each environment *h*, accounting for additional variance, i.e., variation that is not explained by latent common factors [18]*.* The total number of parameters fitted in the FA model is *n*(*k* + 1)-*k*(*k*-1)/2 and may not exceed *n*(*n* + 1)/2, where *n* is the size of **G** matrix from the multivariate model, and *k* is the number of latent common factors. When *k* is equal to 1, the number of parameters fitted in FA is 4(1 + 1) -1(1-1)/2 = 8, compared to 4(4 + 1)/2 = 10 for the multivariate model [19]. The eight parameters are four elements of estimated loading $\left({\hat{\gamma }}_{h}\right)$ and four estimated specific variance $\left({\hat{\psi }}_{h}\right)$ for each environment *h*. The number of factors cannot be higher than 1 in this study.

In ASReml, different types of FA models can be implemented [19]. In this study, we used the extended FA (XFA) [18,19] model, which provides estimates of the **G**_**FA**_ matrix, of loading parameters, of correlations between genetic effects in four environments, and of the latent common factor. The additive genetic variance $\left({\hat{V}}_{A}\right)$ for a certain environment was estimated as: ${\hat{V}}_{A_{h}}={\hat{\gamma }}_{h}^{'}{\hat{\gamma }}_{h}+{\hat{\psi }}_{h}$. The square of the loading parameter indicates the amount of additive genetic effect explained by the latent common factor. A high loading for an environment indicates that the latent common factor explains a large amount of the additive genetic variance in that environment. The percentage of additive genetic variance explained by latent common factors in a specific environment was calculated as: $\%\mathrm{Expl}=\frac{{\hat{\gamma }}_{h}^{'}{\hat{\gamma }}_{h}}{{\hat{V}}_{A}}\times 100$. The covariance of BWH between environments *i* and *j* was calculated as γ^i'γ^j. The *r*_g_ between BWH measured in different environments *i* and *j* was estimated as: $r_{g}=\frac{{\hat{\gamma }}_{i}^{'}{\hat{\gamma }}_{j}}{\sqrt{{\hat{V}}_{A_{i}}\times {\hat{V}}_{A_{j}}}}$.

Initially, the environmental effect common to full-sibs (caused by family rearing until tagging) was included in the model. However, ASReml had difficulty in disentangling genetic (co)variance components from common environmental (co)variance components. The solution was not positive definite and therefore we decided to exclude the common environmental effect from this and all other models in this study.

#### Model comparison

Reaction norm and FA models were compared using Akaike’s information criteria (AIC: [20]) and Bayesian’s information criteria (BIC: [21]). The model with the lowest AIC and BIC indicates the most parsimonious model. All models were kept the same with respect to fixed effects so that they were comparable in terms of REML log likelihood.

### Identification of EV

With a reaction norm model, the EV that provide the best fit to the data will result in the highest log likelihood of the model. In addition, the mean square deviation (MSD) was calculated as the difference between estimated genetic correlations obtained from reaction norm and multivariate (MUV) models and was computed as $\mathrm{MSD}=\frac{\sum_{i=1}^{n}{\left(r_{g_{\mathrm{RN},i}}-r_{g_{\mathrm{MUV},i}}\right)}^{2}}{n}$, where $r_{g_{\mathrm{RN},i}}$ and $r_{g_{\mathrm{MUV},i}}$ are the estimated genetic correlations of BWH between different environments obtained from the reaction norm and multivariate models, respectively. The *i*^th^ genetic correlation was from the same pair of environments for both models and *n* was equal to six, because with four environments there are six genetic correlations. The reaction norm model with the lowest MSD is the model that deviates least from the multivariate model, which indicates that the EV used in that reaction norm model is able to capture the GxE interaction.

The FA model was used as the first step in a two-step approach. The second step consisted of estimating correlations between loadings and means of EV. Pearson (*ρ*_*EP*,*L*_) and Kendall rank (*τ*_*EP*,*L*_) correlations between loading of the latent common factor and EV were calculated to identify the EV that shows the highest correlation with the latent common factor, which is the most likely EV that caused the GxE interaction.

## Results

### Reaction norm model

**Figure 2:**
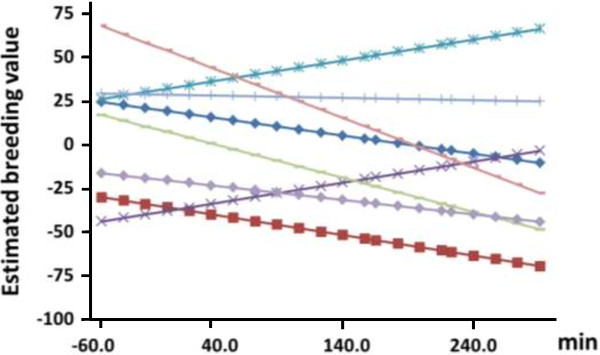
**Estimated breeding values of sires for body weight (y-axis: in grams) against photoperiod (min) using the reaction norm model.** Only eight randomly chosen sires are plotted in this graph to illustrate the degree of re-ranking.

For the reaction norm model, estimates of *r*_g_ of BWH between different environments are in Table 2. For age at harvest, estimates of *r*_g_ varied between 0.57 and 0.99 (MSD = 0.17). For water temperature, estimates of *r*_g_ varied between 0.61 and 0.99 (MSD = 0.19). Estimates of *r*_g_ for Day*Degree were lower and varied between 0.35 and 0.97 (MSD = 0.09). For dissolved oxygen, estimates of *r*_g_ ranged from 0.60 to 0.99 (MSD = 0.14), which was similar to the range of estimates of *r*_g_ for water temperature. For photoperiod, estimates of *r*_g_ ranged from 0.37 to 0.97 (MSD = 0.12). Reaction norm models with day-degree and photoperiod as EV resulted in genetic correlations closest to the multivariate model, which indicates that day-degree and photoperiod were the most important EV that explain the GxE interaction. A plot of the EBV of eight randomly selected sires against photoperiod shows that the GxE interaction was caused by both heterogeneity of additive genetic variance and re-ranking (Figure 2).

**Table 2:** Estimates of genetic correlations of body weight at harvest measured in different environments and mean square deviation (MSD*) between estimates from reaction norm and multivariate models

**Model**	**EV**	**Environment**	**FI**	**PE**	**GE**	**MSD**
Reaction norm	Age	NUC	0.99 ± 0.00	0.91 ± 0.03	0.57 ± 0.11	0.17
		FI		0.94 ± 0.02	0.63 ± 0.10	
		PE			0.86 ± 0.04	
	Temperature	NUC	0.97 ± 0.01	0.99 ± 0.00	0.61 ± 0.11	0.19
		FI		0.98 ± 0.01	0.79 ± 0.06	
		PE			0.65 ± 0.10	
	Day*Degree	NUC	0.97 ± 0.01	0.56 ± 0.10	0.82 ± 0.05	0.09
		FI		0.35 ± 0.13	0.66 ± 0.10	
		PE			0.93 ± 0.02	
	Oxygen	NUC	0.75 ± 0.06	0.99 ± 0.00	0.45 ± 0.12	0.14
		FI		0.68 ± 0.08	0.93 ± 0.02	
		PE			0.36 ± 0.13	
	Photoperiod	NUC	0.97 ± 0.01	0.60 ± 0.09	0.96 ± 0.01	0.12
		FI		0.78 ± 0.06	0.87 ± 0.04	
		PE			0.37 ± 0.13	
Factor analytic	Latent	NUC	0.56 ± 0.06	0.36 ± 0.04	0.54 ± 0.06	N.A.
		FI		0.41 ± 0.05	0.61 ± 0.07	
		PE			0.39 ± 0.05	
Multivariate	N. A.	NUC	0.61 ± 0.10	0.25 ± 0.13	0.53 ±0.12	N.A.
		FI		0.40 ± 0.12	0.55 ± 0.12	
		PE			0.49 ± 0.12	

EV = environmental variables; NUC = breeding environment; FI = Freshwater Institute; PE = Peru; GE = Germany; EV = environmental variable; N.A. = not applicable.

* $\mathrm{MSD}=\frac{\sum_{i=1}^{n}{\left(r_{g_{\mathrm{RN},i}}-r_{g_{\mathrm{MUV},i}}\right)}^{2}}{n}$, where $r_{g_{\mathrm{RN},i}}$ and $r_{g_{\mathrm{MUV},i}}$ are the estimated genetic correlation of BWH between different environments from reaction norm (RN) and multivariate (MUV) models.

### Factor analytic model

For the FA model, estimates of *r*_g_ of BWH between PE and NUC (0.36), between PE and FI (0.41), and between PE and GE (0.39) were low, which indicate moderate to strong re-ranking. The estimate of *r*_g_ of BWH between NUC and FI was much lower (0.56) than the estimate obtained through the reaction norm model when the EV was photoperiod (0.97) or Day*Degree (0.97). For photoperiod, the covariance between NUC and FI obtained through the reaction norm model was similar to that obtained through the FA model (1524.3 and 1555.8, respectively), which indicates that the higher estimate of *r*_g_ obtained with the reaction norm model (photoperiod) was mainly caused by a lower *V*_A_ (NUC: 1574 and FI: 1570), as shown in Table 3. In contrast, for Day*Degree as the EV, the reaction norm model gave a high estimate of *r*_g_ due to both a higher covariance between NUC and FI (1931.7) and a lower *V*_A_ (NUC: 1774 and FI: 2221) compared to those obtained through the FA model. Elements of the estimated loading vector $\hat{\gamma }$ were equal to 40.34 for NUC, 38.57 for FI, 30.41 for PE, and 30.70 for GE, which means that the latent common factor explained most of the of *V*_A_ in NUC and FI (Table 3). The proportion of genetic variance explained by the common factor was only 26.20% for PE and $\hat{\psi }$ was high in PE (2606.73), which showed that much of the additive genetic variance was not accounted for by the latent common factor.

**Table 3:** **Estimates of the total genetic variance** ($\left({\hat{V}}_{A}\right)$**), loadings(**$\left(\hat{\gamma }\right)$**), specific genetic variances(**$\left(\hat{\psi }\right)$**), and % genetic variance explained by the latent common factor (%Expl) for each environment**

**Environment**	${\hat{V}}_{A}$_**, MUV**_	${\hat{V}}_{A}$_**, RN, PP**_	${\hat{V}}_{A}$_**, RN, DD**_	${\hat{V}}_{A}$_**, FA**_	$\hat{\gamma }$	$\hat{\psi }$	**%Expl**
NUC	3304	1574	1774	3283	40.3	1656	49.6
FI	2405	1570	2221	2362	38.6	874	63.0
PE	3558	3161	2455	3531	30.4	2607	26.2
GE	1638	1822	1713	1613	30.7	671	58.4

NUC = breeding environment; FI = Freshwater Institute; PE = Peru; GE = Germany; MUV = multivariate model; RN = reaction norm model for photoperiod (PP) and Day*Degree (DD); FA = factor analytic model.

Pearson correlations (*ρ*_*EP*,*L*_) between the latent common factor and the known EV were negative and high for Day*Degree (-0.91), and for age at harvest (-0.86). The Kendall rank correlation (*τ*_*EP*,*L*_) was in agreement with the (*ρ*_*EP*,*L*_) but lower for both Day*Degree (*τ*_*EP*,*L*_ = − 0.67) and age at harvest (*τ*_*EP*,*L*_ = − 0.67) (Table 4). Water temperature was moderately correlated with the latent common factor (*ρ*_*EP*,*L*_ = 0.50). Dissolved oxygen was weakly correlated (*ρ*_*EP*,*L*_ = − 0.14) or not correlated (*τ*_*EP*,*L*_ = 0.00) with the latent common factor. Photoperiod was positively correlated with the latent common factor (*ρ*_*EP*,*L*_ = 0.32, *τ*_*EP*,*L*_ = 0.33). These results indicate that Day*Degree was the most likely EV responsible for the GxE interaction in BWH.

**Table 4:** Correlations between loadings* and environmental variables for body weight at harvest

Environmental variable**	**Pearson**	**Kendall rank**
Age	−0.86	−0.67
Temperature	0.50	0.33
Day*Degree	−0.91	−0.67
Oxygen	−0.14	0.00
Photoperiod	0.32	0.33

*Obtained from factor analytic model.

**Mean of environmental variable (Table 1).

### Model comparison

With the reaction norm model, the lowest AIC (87645.7) and BIC (87695.3) were obtained for photoperiod, which indicated that it was the best fitted EV, compared to the other EV (Table 5). However, Day*Degree (AIC = 87656.5, BIC = 87706.2) fitted the model similarly well. The best fit was concordant with a lower average estimate of *r*_g_ for either photoperiod or Day*Degree. The AIC (87513.0) and BIC (87528.6) were lower with the FA model than with the reaction norm model, which indicates that the FA model is more parsimonious than the reaction norm model.

**Table 5:** Model comparison between five different reaction norm models, factor analytic model, and multivariate model on body weight at harvest

**Model**	**EV**	**LogL**	**NPar**	**AIC**	**BIC**
Reaction norm	Age	0.0	7	87721.5	87735.1
	Temperature	3.1	7	87715.3	87728.9
	Day*Degree	32.5	7	87656.5	87670.2
	Oxygen	38.8	7	87643.8	87657.5
	Photoperiod	43.0	7	**87635.4**	**87649.0**
Factor analytic	Latent	105.2	12	**87521.0**	**87544.4**
Multivariate	N.A.	114.12	14	87507.2	87534.5

EV = environmental variable; LogL = natural logarithm of likelihood deviated from the smallest value (Age: -43853.7); NPar = number of parameters; AIC = Akaike’s information criterion, BIC = Bayesian’s information criterion; bold letter indicates the lowest AIC and BIC from both random regression and factor analytic models; residual degrees of freedom are equal to 8854 (reaction norm and factor analytic model) and 8853 (multivariate model); N.A. = not applicable.

## Discussion

The aim of this study was to identify the environmental variables (EV) that explain the GxE interaction for body weight at harvest (BWH) of rainbow trout using a reaction norm model and a factor analytic model.

### Identification of environmental variables

To our knowledge, this is the first study that implemented reaction norm and factor analytic models to identify significant EV responsible for GxE interaction in aquaculture. Our findings show that both methods can be used to identify significant EV. However, the reaction norm and FA models identified different significant EV. Based on AIC and BIC, photoperiod gave a slightly better fit with the reaction norm model than Day*Degree, which indicates that photoperiod may also be the most significant EV. However with the FA model, Day*Degree was highly negatively correlated (Pearson correlation: *ρ*_*EP*,*L*_ = − 0.91) with loadings of the latent common factor, which suggests that Day*Degree was the most significant EV. Both the reaction norm and FA models indicate that Day*Degree is an important EV and that it is less likely that temperature is responsible for the GxE interaction. However, the power to identify EV is limited due to having only four environments.

Identification of environmental variables that explain GxE interaction has been studied using different methods. In Guernsey cows from four different countries, among the 15 environmental variables that were studied using a random regression model, nine indicated the presence of GxE interaction (estimates of *r*_g_ varied between 0.85 and 0.98) [4]. By calculating genetic correlations between animals from opposite ends of environmental gradients, Zwald et al. [6] reported that seven of 13 EV caused genetic correlations to deviate from unity (*r*_g_ = 0.79 to 0.90).

Identification of significant EV that cause GxE interaction is valuable because the information can be used to reduce GxE interaction before optimization of a breeding program. Optimization of a breeding program may be more expensive than changing the significant EV so that they are similar across environments, thereby reducing GxE interaction, because of the possible need to establish multiple sib-testing stations or environment-specific breeding programs. However, changing EV to be similar across environments may be expensive or impossible for some farmers or producers, e.g. in the case of sea water temperature. It may be more reasonable to manipulate EV in the breeding environment (NUC) rather than across all diverging production environments (FI, PE, and GE). However, the decision on which EV to manipulate in the NUC will depend on the relative economic importance of the corresponding production environments for which this EV is relevant. A reduction in genotype re-ranking across environments would lead to an increase of genetic gain of BWH in the production environments but the extra profit that this generates may be offset by the extra costs of EV manipulation. Finding the significant EV is also of biological interest, because it provides evidence for environmental sensitivity of growth in rainbow trout. Artificial selection will target those fish that perform best in the stable and controlled environment in which selection is usually done. This could lead to increased environmental sensitivity across multiple environments [22,23]. The elevated sensitivity develops as a logical consequence of re-ranking GxE interaction and/or when genetic variation in the selected environment is higher than in the non-selected environments. Higher sensitivity to environments may have negative consequences, such as reduced fitness and poor animal health in challenging environments [24]. Alternatively, selection for high growth performance in a challenging environment may lead to more robust and better adapted fish to commercial production environments, thus reducing the detrimental side effects on, or even improving, survival or disease resistance [14,25].

Previous studies have shown that photoperiod is one of the major factors that influence growth in rainbow trout [26-28]. In general, longer day length tends to increase growth rate. Taylor et al. [27] found that rainbow trout exposed to a light to dark hours (L:D) rhythm of 18:6 grew significantly faster than rainbow trout exposed to L:D = 8:16, and expressed significantly higher circulating levels of insulin-like growth factor-I (IGF-I) hormone. This hormone is positively correlated with growth rate in rainbow trout [27]. These observations support the idea that photoperiod may cause the significant GxE interaction for growth if genetic variation in sensitivity to photoperiod exists. The direction of change in day length in Peru is opposite to that in the other locations. The light rhythm can be manipulated in aquaculture production. Manipulation of photoperiod by placing lamps under or above the water is becoming common practice to enhance growth and delay sexual maturation in Atlantic salmon and rainbow trout [28]. Therefore, it may be possible to reduce the GxE interaction due to different photoperiods.

Day*Degree is a combination of two factors: days to harvest, which determine the length of the rearing period, and average water temperature. Differences in Day*Degree between environments may result from differences in age or temperature, or both. It is easy to adjust age at harvest so that it is the same across environments, to reduce the observed re-ranking. However, commercial market weights differ between countries, and thus the age differences must be maintained. Most of the production of rainbow trout occurs in fresh and sea water net pens, ponds or raceways, in which temperature control is difficult.

### Model comparison

In this study, the most significant EV was identified using the following criteria with the reaction norm model: the EV that best fitted the data based on AIC and BIC and the EV that resulted in the lowest mean square deviation (MSD) between estimates of *r*_g_ from the reaction norm and multivariate models. Due to the lack of continuous gradients within environments, the reaction norm model resembled a model with categorical EV. The reaction norm model would pinpoint the EV more efficiently if the EV were measured on a more continuous scale (e.g. more environments or treatments). The factor analytic model is frequently used in plant breeding, for example in multi-environment trials to analyse data on variety testing [29]. The factor analytic model is the random version of a model with additive main effects and multiplicative interaction (AMMI) [30-32]. Recently, it was suggested that the factor analytic model was useful to estimate GxE interaction in animal breeding [18]. The factor analytic model was used in international sire evaluations to reduce the number of parameters to be estimated, compared to estimating the full genetic variance-covariance matrix between countries [33]. Our study used a two-step factor analytic model to identify the environmental variable responsible for the GxE interaction. The advantage of using a factor analytic model is the ability to analyse latent common factors, which can be correlated to known EV [7], as shown in our study. The latent common factor can be regarded as either a single factor or a composite of environmental factors, because several environmental factors may contribute to the GxE interaction between environments.

The latent common factor in this study explained genetic variance in body weight at harvest differently between environments. For instance, the latent common factor explained only 26.2% of the total additive genetic variance for BWH recorded in PE but 63% for BWH recorded in FI. These differences in the percentage of explained additive genetic variance indicates the presence of GxE interaction. In all environments, the percentage of additive genetic variance was less than 100%, which indicates that more than one latent common factor explained the GxE interaction. Due to the limited dimension of the **G** matrix, the second latent common factor could not be studied, which would require, e.g., a 5×5 matrix and that the experiment is conducted in at least five farms or locations. The second latent common factor is expected to explain mainly additive genetic variance in PE because common factors are orthogonal and *V*_A_ in the other environments was mainly explained by the first latent common factor. Moreover, with a limited number of environments, the correlation between the latent common factor and the EV may not be accurate and therefore no solid conclusions can be made about EV that explain the GxE interaction. Therefore, it is recommended that a higher number of environments are investigated in future research on GxE interaction.

Based on AIC and BIC, the factor analytic model was more parsimonious than the reaction norm model, which indicates that the factor analytic model was the most suitable for our data set. This model is suitable when the experiment does not have multiple farms per environment, and to study latent common factors across environments. With more than five environments, multiple latent common factors can be studied [32].

As an alternative to the reaction norm or factor analytic models, a hybrid between these two models can be used to capture the GxE interaction and to identify EV. By adding the environment-specific random effect from the FA model to the first order reaction norm model, we can quantify how much of the GxE interaction between environments is explained by the reaction norm on the EV, without the need to compare to the multivariate model. For Day*Degree, the preliminary results from such a hybrid model indicated a better goodness of fit (AIC = 87520.0 and BIC = 87598.0; results not shown) than the original reaction norm model (AIC = 87656.5 and BIC = 87670.2), as expected. The MSD from the hybrid model was equal to 0.005, which implies that the hybrid model could capture all the GxE interaction present between environments like the multivariate model. This contrasts with the reaction norm model (MSD = 0.09), which deviated more from the multivariate model. For photoperiod, the hybrid model also had a better goodness of fit (AIC = 87524.2 and BIC = 87539.8; results not shown) than the reaction norm model (AIC = 87635.4 and BIC = 87649.0). Thus, the hybrid model is potentially useful to study GxE interaction and to identify EV.

The common environmental effect was excluded from both reaction norm and factor analytic models. In our previous study with the same data, this effect explained a small proportion of the phenotypic variation (*c*^2^ ≤ 0.04) [14]. In general, the common environmental effect is high in early life stages but decreases over time. In addition, when a common environmental effect was fitted, ASReml could not easily find positive definite solutions and a converged log-likelihood. This is probably due to the difficulty in disentangling genetic (co)variance components from common environmental (co)variance components. Additive genetic variances may be inflated when ignoring common environmental effects [14,34] and might result in biased estimates of the genetic correlation. However, the bias in the genetic correlation would depend on biases in the additive genetic covariance and in the additive genetic variances, and might be small if all components are proportionally affected. Practical factorial mating designs and/or multigenerational data may be more efficient in separating genetic and common environmental (co)variance components. This should be taken into account in future studies on GxE interaction.

## Conclusions

Day*Degree and photoperiod were identified as environmental variables causing a strong GxE interaction for BWH in rainbow trout across four environments. Both the reaction norm and factor analytic models can contribute to the identification of environmental variables responsible for the GxE interaction. A factor analytic model is preferred over a reaction norm model when limited information on the variation of EV between farms is available.

## Competing interests

The authors declare that they have no competing interests.

## Authors’ contributions

PSL, HK and AK designed and conducted the experiment. PSL mainly analyzed the data and drafted this manuscript. HAM contributed to the theory and methods used in this manuscript. HAM also contributed to the data analysis and writing of the manuscript. HK and AK contributed to the discussions on theory, data analysis, the structure of the manuscript, and writing of the manuscript. All authors read and approved the final manuscript.
